# Robust genetic transformation of sorghum (*Sorghum bicolor* L.) using differentiating embryogenic callus induced from immature embryos

**DOI:** 10.1186/s13007-017-0260-9

**Published:** 2017-12-08

**Authors:** Srinivas Belide, Thomas Vanhercke, James Robertson Petrie, Surinder Pal Singh

**Affiliations:** CSIRO Agriculture and Food, Canberra, 2601 Australia

**Keywords:** Lipoic acid, Nodular structures, Particle bombardment, Geneticin, Flow cytometry, Ploidy analysis

## Abstract

**Background:**

Sorghum (*Sorghum bicolor* L.) is one of the world’s most important cereal crops grown for multiple applications and has been identified as a potential biofuel crop. Despite several decades of study, sorghum has been widely considered as a recalcitrant major crop for transformation due to accumulation of phenolic compounds, lack of model genotypes, low regeneration frequency and loss of regeneration potential through sub-cultures. Among different explants used for genetic transformation of sorghum, immature embryos are ideal over other explants. However, the continuous supply of quality immature embryos for transformation is labour intensive and expensive. In addition, transformation efficiencies are also influenced by environmental conditions (light and temperature). Despite these challenges, immature embryos remain the predominant choice because of their success rate and also due to non-availability of other dependable explants without compromising the transformation efficiency.

**Results:**

We report here a robust genetic transformation method for sorghum (Tx430) using differentiating embryogenic calli (DEC) with nodular structures induced from immature embryos and maintained for more than a year without losing regeneration potential on modified MS media. The addition of lipoic acid (LA) to callus induction media along with optimized growth regulators increased callus induction frequency from 61.3 ± 3.2 to 79 ± 6.5% from immature embryos (1.5–2.0 mm in length) isolated 12–15 days after pollination. Similarly, the regeneration efficiency and the number of shoots from DEC tissue was enhanced by LA. The optimized regeneration system in combination with particle bombardment resulted in an average transformation efficiency (TE) of 27.2 or 46.6% based on the selection strategy, 25% to twofold higher TE than published reports in Tx430. Up to 100% putative transgenic shoots were positive for *npt*-*II* by PCR and 48% of events had < 3 copies of transgenes as determined by digital droplet PCR. Reproducibility of this method was demonstrated by generating ~ 800 transgenic plants using 10 different gene constructs.

**Conclusions:**

This protocol demonstrates significant improvements in both efficiency and ease of use over existing sorghum transformation methods using PDS, also enables quick hypothesis testing in the production of various high value products in sorghum.

**Electronic supplementary material:**

The online version of this article (10.1186/s13007-017-0260-9) contains supplementary material, which is available to authorized users.

## Background

Sorghum is one of the world’s most important cereal crops grown for multiple uses such as food, fuel, forage and fodder. This versatile crop can tolerate drought, soil toxicities, has the ability to grow in a wide range of temperature and climate conditions. The inherent capability to produce high yields with less water & other inputs made sorghum an important staple crop in Africa and is attracting renewed interest globally. The recent rise in popularity of gluten-free diets has also brought new attention to sorghum. Because of its wide uses and adaptation, sorghum is considered an indispensable crop for the survival of humankind [1, 2]. In addition, sorghum has been identified as a prospective bioenergy crop and model for C4 grasses [3, 4]. Further, it has been recently identified as a potential candidate for making environmentally friendly fuels and chemicals, particularly to engineer leaves and stems of this biomass crop to produce more oil instead of starch that offers alternatives to petroleum-based products. To manipulate these complex pathways using genetic engineering approach, highly efficient transformation methods are prerequisite.

Despite several decades of study, sorghum has been widely considered as a recalcitrant major crop for transformation [5], due to tissue culture limitations (accumulation of phenolic compounds), lack of model genotypes, low regeneration frequency and loss of regeneration potential through sub-cultures [6]. As a result, a highly efficient and comprehensive transformation system has remained elusive till date [7]. Particle bombardment and *Agrobacterium*-mediated transformation are two main approaches that have been utilized to obtain transgenic sorghum. The first successful genetic transformation of sorghum using particle delivery system (PDS) was reported using immature embryos with a frequency of 0.08% [8]. Since then several improvements have been reported [9–11] and transformation efficiency ranged from 1 to 7%. However the most significant improvement in transformation efficiency, 20.7% was reported by Liu and Godwin [12] using immature embryos of Tx430. Similarly the first successful report of *Agrobacterium* mediated transformation of sorghum was reported in 2000 [13]. Many modifications to *Agrobacterium* based protocols have been published, achieving transformation frequencies 4.5% [14] and 14% [15]. A notable further improvement in *Agrobacterium* mediated transformation efficiency of sorghum was achieved by adding copper sulfate and 6-benzylaminopurine following infection giving a transformation frequency of 33.2% using immature embryos of Tx430 with super-binary construct [16] but their use is subject to proprietary restrictions and may be expensive. An alternative efficient and cost effective method would be desirable for the improvement of sorghum.

Among different explants used for successful genetic transformation of sorghum (PDS or *Agrobacterium*), immature embryos are the choice of explants over mature seed derived explants (shoot tip) and all the efficient transformation protocols by PDS or *Agrobacterium* largely employed immature embryos [12, 15, 16]. One of the main challenges of using immature embryos directly as explants is the need for continued planting of stock plants to ensure a constant supply of immature embryos which is difficult because restrictive conditions for flowering allows only a small window for collection of suitable embryos [6]. The cost and labour associated with immature embryo isolation is significant especially when large numbers are required to generate hundreds and occasionally thousands of transgenic events. Manual embryo excision also poses ergonomic injury risks when large numbers are required [17]. In addition to these limitations, the donor plant physiological condition has an impact on the quality of the embryo which can influence the callus initiation frequency from the transformed cells. Zhao et al. [13] showed that source of embryos had very significant impact on transformation efficiency.

To overcome some of these problems and to have a reliable and continuous supply of explants for transformation, highly regenerative green tissue from mature seeds of rice [18], immature embryos of barley [19, 20] and wheat [21] have been developed and used for successful transformation. Highly regenerative, green tissues, initiated and maintained on customized media were used to regenerate multiple shoots from a number of cereals and grasses and could be maintained for more than 1 year with minimal loss of regenerability. DNA methylation analyses also indicated that barley plants regenerated directly from these highly regenerative tissues incurred less methylation polymorphism and demonstrated better agronomic performance than those regenerated from non-green embryogenic callus tissues initiated and maintained on 2,4-D alone [22, 23]. However, no similar attempts have been made in generating a green tissue for long term usage in sorghum.

Here, we report a robust transformation method of sorghum using differentiating embryogenic callus (DEC), which circumvented the bottlenecks in the supply of quality immature embryos and their excision. We have used this efficient and reproducible method in large scale transformation to produce high leaf oil lines in sorghum based on our previous genetic strategies developed for tobacco [24]. We have generated more than 800 independent transgenic events by co-bombarding different plasmids (leaf oil constructs) using DEC tissues raised from a couple of hundred immature embryos and utilized for more than a year. Molecular analysis of transgenic lines revealed stable integration and inheritance of transgenes. We also demonstrate for the first time the use of lipoic acid to achieve significant improvements in regeneration and transformation efficiencies of sorghum.

## Methods

### Plant material

Plants of grain sorghum inbred line Tx-430 [25] were grown in a plant growth chamber (Conviron, PGC-20 flex) at 28 ± 1/20 ± 1 °C (day/night) temperature, with a 16 h photoperiod of 600 µmol/s m^2^. Panicles were covered with white translucent paper bags before flowering. Immature embryos were harvested from panicles 12–15 days after anthesis. Immature seeds were sterilized as described by Liu and Godwin [12].

### Media composition and modifications

Media used in the present study are based on MS media [26] ready to use powder (PhytoTechnology Laboratories, M519). The pH of the media was adjusted to 5.8 before sterilization at 121 °C for 15 min. Heat sensitive plant growth regulators, other additives such as copper sulfate (CuSO4), lipoic acid, l-cysteine, ascorbic acid, thidiazuron (TDZ) and geneticin (G418, Sigma) were filter sterilized (0.2 μm) and added to the media after sterilization when the media is about 55 °C. Media used in different steps of DEC tissue induction, transformation and plant regeneration were summarized in Additional file 1: Table S1 and Additional file 2: Table S2.

### Optimization of differentiating embryogenic callus development, maintenance of DEC tissue and plant regeneration

Immature embryos (IE) ranging from 1.4 to 2.5 mm in length were aseptically isolated in laminar flow hood and inoculated onto CIM medium (Additional file 1: Table S1) with (1–5 mg/l) or without the addition of LA, with their scutellum facing upward. For the development of DEC tissue, immature embryos were incubated under fluorescent light of approximately 45–50 µmol/s m^2^ (16 h/day) in a tissue culture room at 24 ± 2 °C. After 3 days of culture, growing root and shoot portions of immature embryos were aseptically chopped and re-inoculated onto CIM medium (Additional file 1: Table S1) and maintained under the same conditions as described above and subcultured every 2 weeks onto the same media (CIM) for 8 weeks. Callus induction frequency was calculated as the number of immature embryos producing calli (~ 5 mm) per total number of immature embryo. For the maintenance of DEC tissue, uniform in size (~ 5 mm diameter) and very similar looking callus (early globular stage) were selected and cultured on CIM. After 2 weeks culture on CIM, grown DEC tissue (up to 10 mm) chopped again into small pieces (~ 5 mm) and cultured on to CIM as described above.

Plant  regeneration from  DEC (uniform in size, ~ 5 mm diameter) was tested on media with different combinations and concentrations of 2,4-D, BAP and TDZ; the preferred combinations of shoot regeneration media (SRM) shown in Additional file 1: Table S1. Regeneration frequency of DEC tissues (2–6 months old) was calculated as number of DEC tissues producing shoots divided by total number of DEC tissues inoculated × 100. Individual shoots, approximately 4–5 cm, were transferred to root induction media (RIM, Additional file 1: Table S1) and cultured for 3–4 weeks. Well rooted shoots (9–10 cm) were transferred to glasshouse and grown to maturity as described. The optimized conditions of plant regeneration from DEC induced from immature embryo used for PDS mediated transformation experiments.

### Flow cytometric analysis

Ploidy nature of regenerated sorghum plants from DEC tissue at different stages (5–24 months since induction from immature embryo) and WT (plant raised from seed) was determined using a Partec PAII flow cytometer. Young leaf tissue, 50 mg from WT and regenerated plants were cut into small pieces using razor blade in a petri dish in 1 ml of Galbraiths buffer (45 mM MgCl_2_, 20 mM MOPS, 30 mM sodium citrate 0.1% (v/v) Triton X-100, pH adjusted to 7 using 1 M NaOH). Homogenate was mixed several times with a pipette and the nuclei suspension was filtered through 38 µm nylon cloth into 1.5 ml Eppendorf tube and centrifuged at 1000 rpm for 2.5 min. Around 800 µl of supernatant was removed leaving 200 µl of homogenate, to which 10 µl propidium iodide solution (1 mg/ml, Sigma) was added and mixed gently, then transferred to flow cytometer tube, incubated at room temperature for 1 h and analysed. 2C DNA content was calculated based on the value of the fluorescence intensity of G1 peaks for both the internal standard (WT) and the sample. The ploidy level and DNA content of the unknown samples were calculated as follows:

Ploidy Nuclear DNA content = mean position of sample peak/mean position of standard peak × DNA content of the standard. The nuclear 2C DNA content of sorghum is reported to be 1.74 pg [27].

### Kill curve with geneticin

A geneticin kill curve experiment was carried out using 8 week old differentiating embryogenic callus (~ 5 mm in diameter) on selective CIM media containing geneticin (G418, Sigma) at 0, 15, 25, 35 and 45 mg/l. Sixteen green calli per plate with three replicas per concentration were cultured for 6 weeks (sub cultured on the same media for every 2 weeks). After 6 weeks of culture, the number of calli surviving was recorded.

### Plasmids

The *uidA*/*bar* vector (Additional file 3: Figure S1-A) was used in experiments to optimize critical parameters by measuring transient gene expression in the sorghum DEC tissues. The *uidA* gene was under the regulatory control of a maize polyubiquitin promoter (Ubi) and an *Agrobacterium tumefaciens* octopine synthase polyadenylation/terminator (*ocs* 3′) sequence. The *bar* gene was also under the regulatory control of a Ubi promoter and terminated with an *Agrobacterium* nopaline synthase 3′ regulatory sequence (nos) [28]. Another binary plasmid (pBSV003) containing *nptII* gene, driven by maize ubiquitin (*Ubi1*) promoter and terminated by nos (Additional file 3: Figure S2-B), was used in stable transformation of sorghum. Plasmid DNA was isolated using Zymopure™ Maxiprep kit as described by the manufacturer (Zymo Research, CA, USA).

### Transformation by PDS

Approximately 15 uniform DEC tissues (4–5 mm diameter) were placed at the centre of a petri dish (15 × 90 mm), containing CIM-osmotic (CIM-OS) medium (Additional file 2: Table S2) and incubated in dark for ~ 4 h prior to the bombardment. Bombardment was performed [29] with a PDS 1000 He device (Biorad, Hercules, CA, USA). Briefly, plasmid DNA (1 µg/µl) was precipitated on to 0.6 µm gold particles and accelerated with helium pressure using 1350 psi rupture discs at 26–27 Hg vacuum. Post bombardment, DEC tissues were kept on CIM-OS overnight then transferred to pre-selection medium (CIM-PS) and incubated for another 3–4 days. Bombarded calli were incubated under fluorescent light of approximately 95–100 µmol/s m^2^ (16 h/day) in a tissue culture room at 24 ± 1 °C.

### Selection and recovery of one or multiple events from a single bombarded DEC tissue

Bombarded green calli recovered on CIM-pre-selection for 3–4 days, were transferred to selection medium (CIM-G25) and cultured through different steps (Additional file 2: Table S2) until putative transgenic shoots were at least 4 cm and transferred to RIM-G15. In another selection procedure, bombarded green calli recovered on CIM-PS for 3–4 days, were transferred to selection medium (CIM-G25) by splitting into two equal pieces of individual callus (Additional file 4: Figure S2) as described by Yukoh and Toshihiko [30].

### DNA extraction and PCR

Genomic DNA was isolated from young leaves of putative transgenic shoots as described by Mieog et al. [31] with minor modifications. Briefly, freeze dried leaf tissues (1 cm^2^) were crushed in 96-well plate with stainless steel ball bearings using a tissuelyser-II (Qiagen). 375 µl of DNA extraction buffer (0.1 M Tris–HCl pH 8.0, 0.05 M EDTA pH 8.0, 1.25% SDS) was added to the crushed tissue. The mixture was then incubated at 65 °C for 1 h after which 187 µl of 6 M ammonium acetate was added and incubated at 4 °C for 30 min. The samples were centrifuged at 3000 rpm for 30 min at room temperature. 340 µl of supernatant was recovered in a new 96 deep-well plate containing 220 µl of isopropanol for precipitation of the DNA. The DNA was rinsed once with 70% ethanol, briefly dried and dissolved in 220 µl sterile distilled water and left overnight at 4 °C. To confirm the presence of *nptII* gene, NPTII-F: 5′-ATGATTGAACAAGATGGATTG-3′ and NPTII-R: 5′-GCTATGTCCTGATAGCGGTCC-3′ primers were used and PCR analysis was performed on genomic DNA of putative transgenic shoots. The thermal profile of the PCR was: initial denaturation at 94 °C for 15 min, 35 cycles of 94 °C for 60 s, 56 °C for 30 s, 72 °C for 1 min, and finally 72 °C for 10 min. Amplified products were size fractionated on 1% w/v agarose gel in TAE buffer. Gel electrophoresis was carried out at 80 volts for 40 min before DNA bands were visualized with a BioRad QuantiOne UV transilluminator and software.

### Transgene copy number analysis by digital droplet PCR

Transgene copy number was determined by digital droplet PCR [32] using primer and probes specific for the *nptII* selectable marker and sorghum *enol*-*2* (Additional file 5: Table S3) reference gene [33]. *Bam*H1 and *Eco*R1 digested genomic DNA was added to ddPCR mastermix at concentrations between 20 and 120 ng of DNA per 25 µl PCR reaction. Final concentrations of Sigma primers and IDT probes in the reaction were 400 nM and 200 nM, respectively. Droplets were generated using a Droplet Generator QX200 (Bio-Rad, Australia) following the manufacturer’s instructions. 40 µl of droplets in oil emulsion was transferred to a 96-well plate and loaded in a C1000 Thermal Cycler (Bio-Rad, Australia). The PCR program consisted of 95 °C for 10 min, 40 cycles of 94 °C for 30 s and 59 °C for 1 min, followed by 98 °C for 10 min, with a 2.5 °C/sec ramping at each step. After amplification, plates were loaded on to the QX100 Droplet Reader (Bio-Rad, Australia) for the detection of amplicons in individual droplets. Data analysis was performed using the Quanta soft software (version 1.7.4.0917; Bio-Rad, Australia).

### Transgene inheritance study

Five PCR confirmed T0 lines containing 2–4 copies of *nptII* gene were selected for inheritance of *nptII* gene analysis. At least 21 seeds from each selected line were grown, DNA extracted and PCR performed as described above. Plasmid DNA from pBSV003 used as positive control and DNA from Tx430 (WT) used as negative control.

### Experimental design and analysis of data

DEC tissue induction, regeneration and transformation experiments were conducted by complete randomized block design and each experiment had at least 15 explants with three replicas. Comparisons between treatments were analysed by One Way Analysis of Variance (ANOVA) using Sigmaplot (v13) by the Holm–Sidak method [34]. Differences were considered significant at the 5% level.

## Results

### Effect of α-lipoic acid on DEC tissue induction frequency, quality and yield

**Fig. 1:**
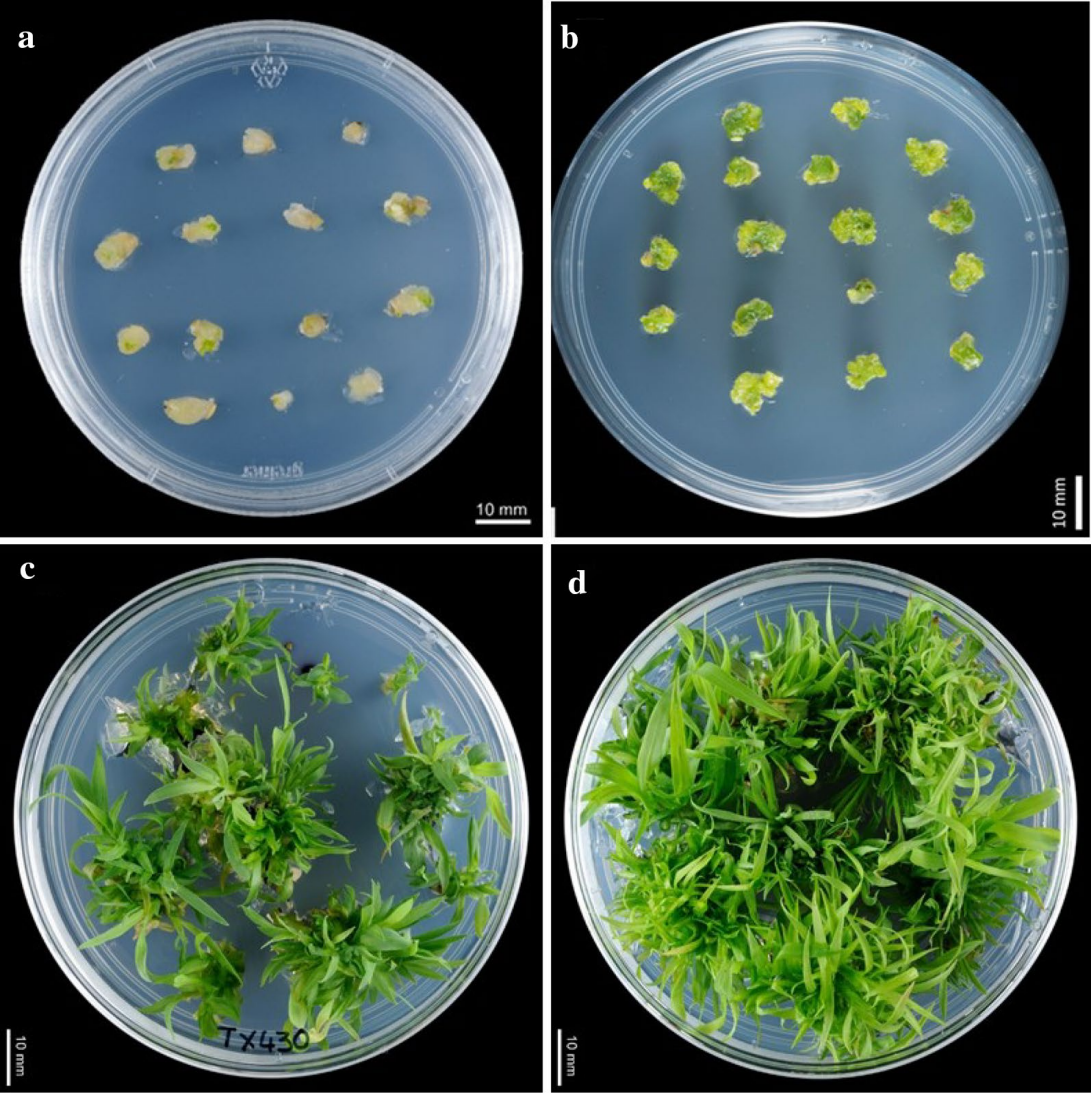
Generation of DEC tissue and plant regeneration from immature embryos of sorghum. **a** Callus initiation from embryo (4 week old), **b** embryogenic callus with nodular structures (at least 8 weeks old), **c**, **d** shoot induction without and with LA in SIM and SRM respectively

Immature embryos (IE) ranging from 1.4 to 2.5 mm in length (from one panicle) were cultured on CIM medium (Additional file 1: Table S1) in the absence or presence of LA (1–5 mg/l). Within 4 weeks on CIM, immature embryos formed callus which is pale yellow to light green in colour (Fig. 1a). After 8 weeks of culture, including subculture every 2 weeks onto the same medium, DEC tissue induction frequency and quality were evaluated. Small (1.4–2 mm) immature embryos cultured on CIM with LA (1 mg/l) had greater DEC tissue induction frequency (significant at *P* < 0.01) with improved quality of nodular structures compared to CIM without LA. Improved quality refers to the visual appearance of the nodular structures, their colour being soft green to darker green (Fig. 1b) and also the relative amount of tissue (at least 50%) that had started to develop into globular stage somatic embryos. The mean callus induction frequency with larger embryos (> 2 mm) on CIM with LA (1 mg/l) or without LA were not significant (*P* = 0.1). Callus from immature embryos (1.4–2 or > 2 mm) cultured without LA were pale yellow to green with less nodular structures and also less globular stage embryos compared to CIM with LA (Table 1). There was no significant further improvement observed in DEC tissue induction frequency and quality of the calli at higher concentrations of LA (2–5 mg/l, data not presented).

**Table 1:** Comparison of DEC tissue induction frequency and quality with and without LA in callus induction medium (CIM)

Immature embryo size (mm)	Parameter	Medium	
		CIM	CIM +1 mg/l LA
1.4–2	DEC tissue induction frequency	61.3 ± 3.2	79.0 ± 6.5*
	DEC tissue quality	Average quality	High quality^a^
> 2	DEC tissue induction frequency	52.3 ± 3.1	59.0 ± 5.5**
	DEC tissue quality	Low quality	Good quality

Values are the means with standard deviation (SD)

* Significant at *P* < 0.013

** Not significant

^a^Refer text for more description

Significant differences in callus induction frequency and quality were observed with and without the addition of LA in CIM, hence we assessed the ready-to-use callus yield from 45 IEs (1.4–2.0 mm, three replicas of 15 each), cultured onto CIM (Additional file 1: Table S1) with and without the addition of LA (1 mg/ml) as described above. After 4–12 weeks of culture, the number of DEC tissues obtained were counted and presented (Additional file 6: Table S4). The number of DEC tissue pieces (~ 5 mm) obtained at 12 weeks on CIM with LA was almost double when compared to CIM without LA.

### Maintenance of DEC tissue for long term usage

Uniform in size (~ 5 mm diameter) and very similar looking callus (Fig. 1b) were selected and maintained on CIM by subculturing every 2 weeks for year or more. At the very end of 2 week subculture cycle on CIM, it was observed the differentiation of nearly half of the callus cells into globular stage embryos (Additional file 7: Figure S3-A). These globular embryos subsequently swollen and very became large (Additional file 7: Figure S3-B) and enlarged in size (Additional file 7: Figure S3-C, D). It was never observed the conversion of globular shaped embryos into other advanced stages and no obvious meristem or mini shoots observed as long as they are cultured on CIM. This continuous proliferation of green cells or early globular embryos could be due to the balance of exogenous and endogenous hormones particularly 2,4-D and very low concentration of BA including low intensity of light.

### Effect of α-lipoic acid on regeneration efficiency and number of shoots/DEC tissue

Uniform ready-to-sue DEC tissues induced on CIM medium without LA were transferred to shoot induction medium (SIM, Additional file 1: Table S1) without LA and cultured for another 2 weeks. Tissues with induced shoot buds were then transferred to shoot regeneration medium (SRM) without LA and again cultured for 2 weeks (Fig. 1c). Similarly uniform ready-to-use DEC tissues induced on CIM medium with added LA were transferred to shoot induction medium (SIM, Additional file 1: Table S1) with LA and cultured for another 2 weeks. Tissues with induced shoot buds were then transferred to shoot regeneration medium (SRM) with added LA and again cultured for 2 weeks (Fig. 1d). Tissues including the shoots were again cultured on hormone free medium (SOG, Additional file 1: Table S1) with or without LA for another 2 weeks based on the CIM and SRM media composition particularly LA. Calli/shoots induced on CIM/SIM with LA were transferred to SOG with LA and those induced on CIM/SIM without LA transferred to SOG without LA. Use of the shoot regeneration medium with the combination of BAP and TDZ was superior compared with use of BAP only (data not presented). The regeneration frequency, number of shoots/DEC tissue for different ages of DEC tissue are presented in Table 2.

**Table 2:** Effect of lipoic acid on regeneration frequency and number of shoots from DEC tissue

DEC tissue age in months (from immature embryo isolation)	Regeneration frequency (%)		Number of shoots/callus	
	No LA in SIM and SRM	LA in SIM and SRM	No LA in SIM and SRM	LA in SIM and SRM
2	92.3 ± 3.05	97.6 ± 2.5	22.0 ± 3.6^a^	40.0 ± 4.5^b^
4	86.0 ± 2.0*	97.0 ± 2.6*	16.0 ± 2.0^c^	40.0 ± 1.5^d^
6	81.0 ± 1.0**	94.3 ± 2.0**	14 .0 ± 2.0^e^	33.0 ± 2.5^f^

Values are the means with standard deviation (SD). Different letters in the row, in calculation of number of shoots/calli is significant (P < 0.050)

* Significant at P = 0.004

** Significant at P ≤ 0.001

### Relative DNA content of regenerated plantlets from different age callus lines

The relative DNA content as determined using flow cytometer (FCM) in regenerated plants derived from different callus lines (5 months–2 years) revealed that nearly all the plants were diploid and DNA content was comparable to WT seed derived ~ 3 weeks old plant (Additional file 8: Table S5). The differences in DNA content between WT and regenerated lines was not significant (P = 0.19) by one-way ANOVA except plants regenerated from callus line CL-13. G2/G1 ratio of WT and regenerated plants from different callus lines was less than 2.0 and there is no significant difference of this ratio in WT and regenerated plants. Histograms of relative nuclear DNA content of leaves from WT and regenerated plants from DEC tissues did not show any distinct G0/G1 peaks (Additional file 9: Figure S4). Plants regenerated from 2 year old callus lines (CL9 and CL10) shown normal fertility and seed setting.

### Geneticin kill curve

DEC tissues began to display geneticin stress symptoms on selective callus induction media (CIM with geneticin) within 3 weeks of culture. DEC tissues without geneticin (G0) in media showed continued cell proliferation and grew healthily without any loss of calli. After 6 weeks on CIM media with different concentrations of geneticin (15, 25, 35 and 45 mg/l), DEC tissues showed differential growth responses (Additional file 10: Table S6). After 6 weeks on CIM with G25 and 35 mg/l, most of the calli turned dark brown colour with no signs of survival. Very few calli (2–6) survived with dead patches, hence 25 mg/l of geneticin  were used in the callus induction media for 2 cycles (2 weeks each) and 1 cycle of 35 mg/l geneticin in shoot induction media was chosen as the selection regime. Different concentrations of geneticin in CIM (25 mg/l) and SIM (35 mg/l) were found to be optimal in preventing the growth of non-transformed cells.

### Effect of l-cysteine and ascorbic acid on recovery of post-bombarded tissues and GUS gene expression

DEC tissues bombarded with plasmid containing the *uidA*/*bar* gene (Additional file 3: Figure S1-A) were transferred to CIM pre-selection media and cultured for 3–4 days with or without addition l-cysteine (50 mg/l) and ascorbic acid (15 mg/l). Without the addition of these antioxidants in pre-selection medium, a few of the bombarded tissues turned brown and lost the ability to grow further. After 3–4 days on pre-selection medium (with or without AA, L-Cyst) the bombarded tissues were subjected to GUS staining and viewed under a microscope to count the distinctive blue (GUS positive) foci. Strong GUS gene expression was observed in all bombarded DEC-tissue with at least 7 foci/callus. The inclusion of the two antioxidants in the pre-selection medium further improved the efficiency of the transformation (at least 20 foci/callus) as shown by the transient expression of the GUS gene (Additional file 11: Table S7). Moreover, distinctive GUS positive spots were observed all over the tissue rather than clustered. This observation encouraged to split the bombarded tissues into two equal parts (before selection) in later experiments (Additional file 4: Figure S2).

### Selection and regeneration of transgenic plants

**Fig. 2:**
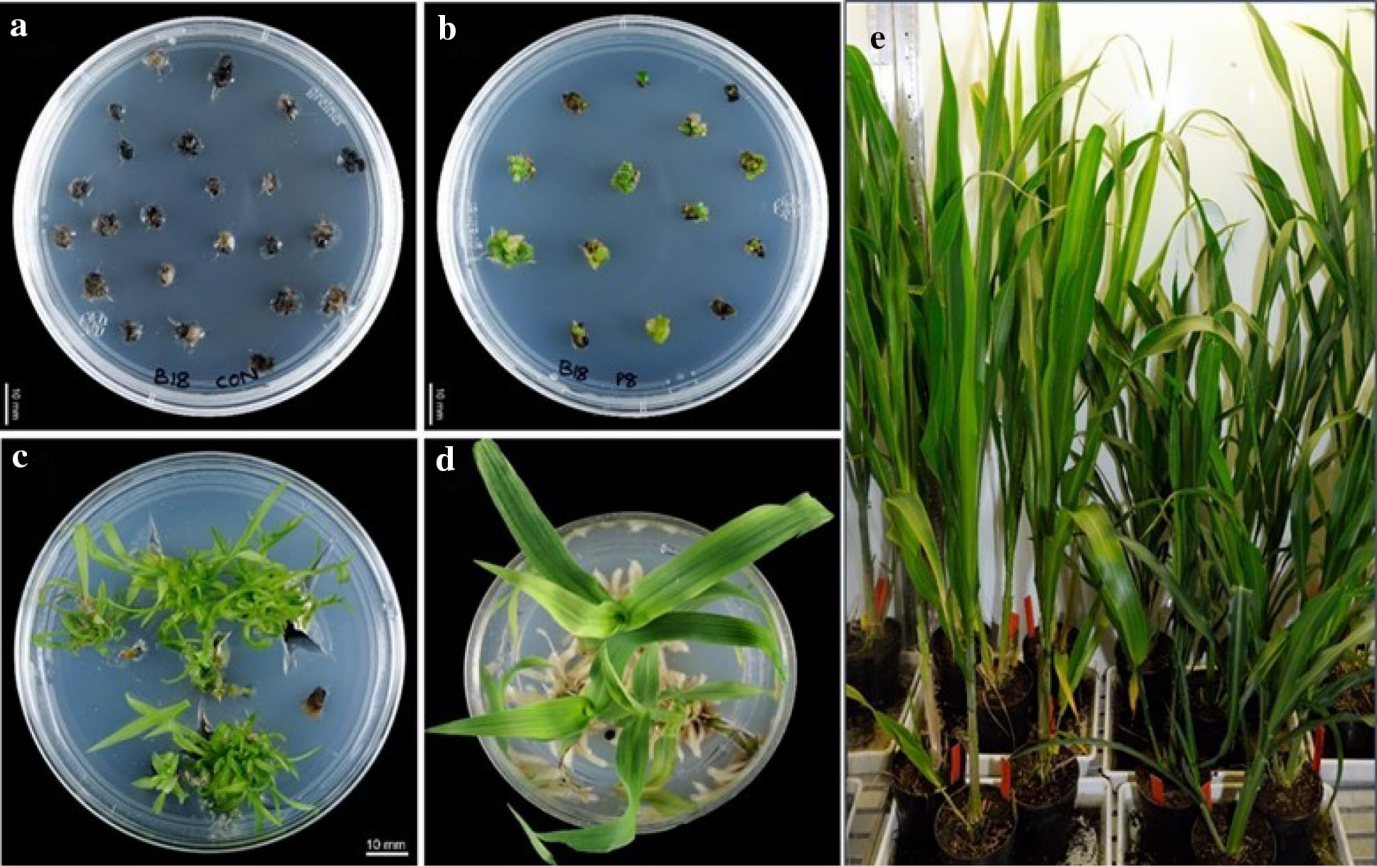
Selection and regeneration transgenic sorghum plants. **a** Non-transformed (control) calli on CIM + G25, **b** Transformed calli on CIM + G25, **c** shoot regeneration from transformed callus, **d** putative transgenic shoot in root induction media, **e** PCR confirmed 2^~^ months old transgenic plants in plant growth chamber

Following bombardment and 3–4 days culture on pre-selection media without selective agent (Geneticin), the bombarded tissues had increased in size from ~ 5 mm to 6–7 mm. These tissues were transferred to CIM containing 25 mg/l geneticin, and cultured for 4 weeks (2 cycles). Each bombarded tissue along with non-bombarded tissue as control (Fig. 2a, b) cultured through the steps described in Additional file 2: Table S2 until putative transgenic shoots were at least 4 cm (Fig. 2c, d). Shoots obtained from a bombarded tissue were cultured and grown in a single media plate for calculation of the transformation efficiency. Though we obtained more shoots (at least 5) from a single selected callus, all the shoots were considered as clones from that callus. Positive transformation was confirmed by PCR on plant genomic DNA isolated from shoot samples, showing the presence of the selectable marker gene. Transformation efficiency (TE %) was calculated as total number of independent events regenerated divided by total number of DEC tissues bombarded. The average transformation efficiency with pBSV003 was 27.2% (Table 3) with this selection regime. This equals to a 23% improvement compared to the previously published method [12] using Tx430 immature embryos by PDS.

**Table 3:** Transformation efficiency of sorghum (Tx430) DEC tissues derived from immature embryos

Number of DEC tissues originally bombarded	Method of selection and subculture	Total number of independent transgenic events regenerated	Transformation efficiency (%)
150	Tissues split into two equal parts, totalling 300 callus pieces	140	46.6
180	Tissues cultured as it is (no split), totalling of 180 pieces	49	27.2

### Regeneration of multiple events from a single bombarded DEC tissue

The average number of GUS foci observed in DEC tissue cultured on CIM with l-cysteine and ascorbic acid was around 27 per tissue and scattered all over the tissue. In addition to this, multiple shoots were regenerated from a single DEC tissue including geneticin selected tissues, which prompted us to find a better selection strategy to obtain multiple events from a single DEC tissue. To explore this, some tissues were split into two approximately equal pieces (Additional file 4: Figure S2) and cultured as described. Shoots obtained from a split tissue maintained separately for calculation of the transformation efficiency. Though we again obtained more shoots (> 5) from a single selected split callus, all the shoots were considered as clones from that split callus. The average transformation efficiency by this split method with pBSV003 was around 46.6% (Table 3). This TE is more than double compared to earlier reports in Tx430 [12] using PDS or better than using a *Agrobacterium* mediated super binary construct [16], while using a more flexible DEC tissue explant.

### Molecular analysis of T0 plants

**Fig. 3:**
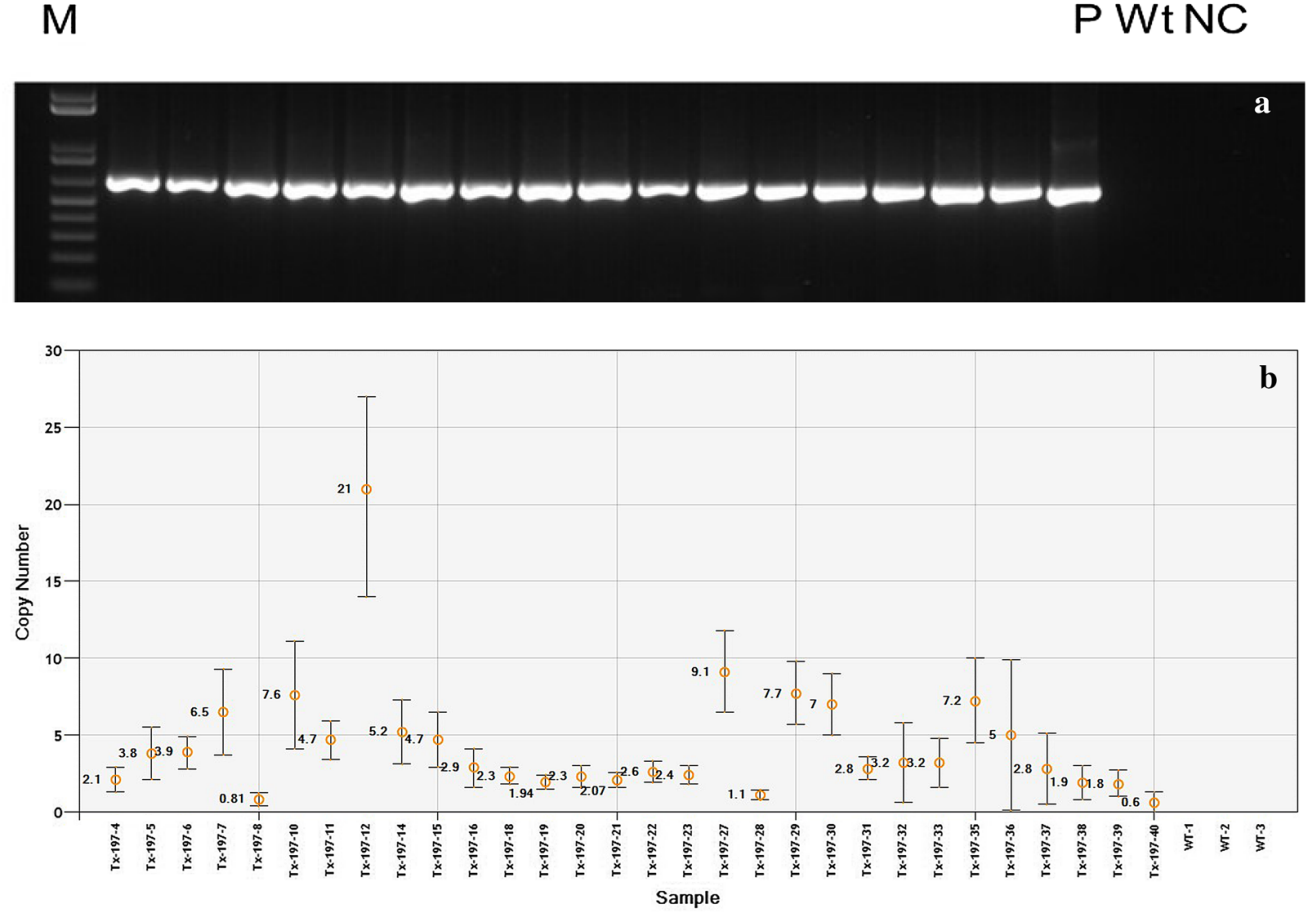
Molecular confirmation of stable T0 transformants. **a** PCR detection of *nptII* transgene in randomly selected lines. Lanes from left to right: M = 1 kb plus DNA ladder, 2–18 transgenic lines, *P* plasmid DNA, *Wt* WT Tx430 DNA, *NC* water control, **b** copy number of *nptII* gene in 30 independent T0 transgenic plants analysed by digital droplet PCR (Error bar represents standard error in reading the copy number)

DNA from 20 randomly selected putative transformants bombarded with pBSV003 (Additional file 3: Figure S1-B) were subjected to PCR analysis to confirm the presence of *nptII* gene. All the putative transformants were positive for *nptII* transgene and with the expected 750 bp amplicon (Fig. 3a) from an internal segment of the gene. In WT DNA, no band was detected. The presence of *nptII* transgene in all the putative transformants indicates the geneticin selection was suitable and effective for DEC tissues. Transgene copy number was analysed by ddPCR with two primers, one designed to target *nptII* (transgene) and other to target endogenous reference gene i.e., *enol*-*2* [33] which is present in sorghum as two copies. Variation in copy number of transgene is determined by calculating the concentration of reference and transgene in 30 randomly selected independent T0 events. Eight of 30 transgenic events displayed less than 2 copies (26.6%), while around 26% of events showed 2–3 copies per genome. The remaining transgenic events (48.3%) were predicted to contain more than 3 copies per genome (Fig. 3b).

### Progeny segregation

PCR screening was used on T1 plants to confirm the inheritance *nptII* transgene. Independent events with 2–3 copies confirmed by dPCR (T0) were selected and Chi square test used to confirm the Mendelian inheritance ratios by screening NPTII positive and negative plants. Out of five events analysed, three events (197-4, 197-6 and 197-18) have shown no significant deviation from Mendelian 3:1 ratios (Table 4). Interestingly copy number of these three events are more than one (Fig. 3b) but the 3:1 segregation ratio suggesting an integration may be at a single locus. On the other hand events 197-20, 197-21 shown deviation from Mendelian ratio and a complex segregation pattern. This segregation pattern also suggests that bombarded DEC explants not leading to any chimeric transgenic plants. Fertility and seed setting was also normal in majority of the transgenic plants with pBSV003, except event 197-6, 197-7 has low seed set and 197-23 no seed set.

**Table 4:** Transgene (*nptII*) segregation analysis of T1 plants

Line ID	Total plants analysed	T1 segregation (positives:negatives)	Chi square value	Copy number by dPCR in T0 plants
Tx197-4	24	17:7	0.12	2
Tx197-6	24	18:6	0.50	3.9
Tx197-18	24	16:8	0.03	2
Tx197-20	24	21:3	3.12*	2
Tx197-21	21	18:3	2.28*	2.6

* Event with significant difference from 3:1 segregation ratio

## Discussion

The continuous supply of high quality immature embryos for transformation is labour intensive, expensive and require considerable greenhouse space. In addition, transformation efficiencies are also influenced by environmental conditions, i.e. light and temperature [13, 15]. Despite these challenges, immature embryos remain the predominant explant for transformation of sorghum because of their success rate and also due to non-availability of other dependable explant without compromising the transformation efficiency. Shoot apices [35] and shoot meristems [10] have been used as alternative explants to alleviate the challenges with supply of immature embryos, however the transformation efficiency remains low with seedling-based explants compared to immature embryos. An explant with robustness and free of environmental factors can aid in large-scale transformation experiments for quick hypothesis testing in ambitious metabolic engineering projects. Here we have developed a robust regeneration and genetic transformation system using DEC tissue induced from immature embryos that can be routinely maintained for more than a year. Our method yielded up to 46.6% transformation efficiency using particle bombardment which is more than double compared to the best published PDS method in sorghum Liu and Godwin [12] or *Agrobacterium* mediated transformation with super binary construct [16] while using a convenient DEC tissue explant. The robustness of this method was largely attributed to four crucial factors: (1) Highly competent explant; (2) Improved regeneration system by addition of antioxidants/enhancers; (3) DNA delivery by PDS and (4) Post-bombardment recovery and selection strategy.

Regeneration of a plant from a transformed cell is often considered the greatest bottleneck in many crops and several factors (genotype, explant, media composition etc.) directly influence the effectiveness of a regeneration system. Liu et al. [7], Liu and Godwin [12] reported improved regeneration (83.3%) and transformation efficiencies (20.7%) with optimized media composition by including CuSO4, KH2PO4, l-proline, l-asparagine and plant growth regulators for inbred line Tx430 using immature embryos. We further improved regeneration efficiency of DEC tissue to 100%. Furthermore, DEC tissue can be maintained for more than a year without losing its regeneration potential by using immature embryos only once. In the present study, DEC tissue proved to be an alternative reliable explant for transformation alleviating the problems associated with immature embryo supply. We achieved high genetic transformation efficiency by using transformation-enhancing compound, lipoic acid (LA) with other additives. LA has been reported as most efficacious compound in reducing the browning/death of transformed cells and improving regeneration thus enhancing transformation frequencies in a number of crops i.e., tomato, soybean, wheat and cotton [36, 37]. We hypothised that the LA might be increasing the overall cellular antioxidant capacity of sorghum DEC tissues, thereby promoting the differentiation, proliferation and regeneration of cells from immature embryos. The positive impact of LA was evident from significantly improved (P < 0.01) DEC tissue induction frequency and also the quality of nodular structures obtained with addition of LA. Ready-to-use DEC tissue yield (number) was almost twofold with addition of LA, ensuring the supply of quality explants with minimum efforts. In addition to this, regeneration frequency and number of shoots per callus (0.5 mm) was higher with addition of LA. Post bombardment recovery of DEC tissues on l-cysteine and ascorbic acid was higher compared to no l-cysteine and ascorbic in pre-selection media. These three antioxidants (LA, l-cysteine and AA) might be synergistically helping the post bombardment recovery of transformed cells there by increasing the survival of transformed cells to form callus and shoots. We have previously shown the beneficial effect of l-cysteine and AA in efficient transformation of safflower [38].

The neomycin phosphotransferase (*npt*-*II*) gene has been shown to be efficient as plant selectable marker in sorghum transformation using immature embryos as explants [12]. Selection of transformed cells by geneticin was very efficient in DEC tissues in the present study, evident by up to 100% putative transgenic shoots being positive for *nptII* gene.

This robust transformation system will enhance our capacity to produce quality transgenic events (low copy number and back bone free) and the ability to test new pathways or the modification of existing pathways. Around 53% of the tested shoots had less than 3 copies/genome. We anticipate that single-copy insertion events can be further enhanced by using very low quantity of DNA per bombardment or by physically separating the vector backbone from the expression cassette [39].

Immature embryo mediated genetic transformation of sorghum typically required 7–8 months’ time to establish a T0 plant in glasshouse. Nearly half of the time (3–4 months) is required to grow immature embryos. Isolation and IE preparation for transformation is also labour intensive. But using this method, DEC tissue can be routinely used for more than a year, sustained regeneration potential, accumulation of no gross variation in ploidy makes this method really suitable for routine large scale transformation projects using PDS or *Agrobacterium*.

## Conclusions

Sorghum is one of the top seven grasses identified as a potential feedstock for a lignocellulosic fuel industry and it is also the primary target of the biofuel feedstock community. This plant is being engineered for several improved traits and recently oil accumulation in vegetative tissues. In addition to metabolic engineering needs, genome editing is emerging as a tool to provide novel opportunities to increase the crop productivity but relies on genetic transformation and plant regeneration, which are bottlenecks in the process. This robust genetic transformation method will be just as relevant to gene editing and metabolic engineering needs of sorghum. A critical pragmatic aspect of this method is the use of flexible explant, large number of transgenic events can be regenerated for new traits with less resources and time.

## Additional files

**Additional file 1: Table S1.** Composition of media used in different steps of DEC tissue induction and plant regeneration from sorghum.

**Additional file 2: Table S2.** Composition of media used in different steps of sorghum transformation using DEC tissues by particle bombardment.

**Additional file 3: Figure S1.** Schematic representation of plasmids used for bombardment of sorghum DEC tissue. A-pUbi-BAR, B-pBSV003.

**Additional file 4: Figure S2.** Method of sectioning and culturing bombarded DEC tissue in selection media.

**Additional file 5: Table S3.** List of primers and probes used in copy number detection by ddPCR in transgenic sorghum.

**Additional file 6: Table S4.** Effect of LA on DEC tissue yield from 45 immature embryos (1.4–2.0 mm) of sorghum.

**Additional file 7: Figure S3.** Stages of differentiating embryogenic callus (DEC) induced and maintained on CIM. A, B Initiation of globular stages embryos. C, D Enlargement of globular stages embryos with out further differentiation.

**Additional file 8: Table S5.** 2C values of nuclear DNA content of leaf tissue regenerated from different age callus lines.

**Additional file 9: Figure S4.** Histograms of nuclei extracted from leaf tissue of regenerated plants from different age callus lines and WT seedling. (A) WT leaf tissue from 3 weeks old plant; (B) CL9: 24 months; (C) CL10: 12 months old; (D-E) CL-11 and CL12: 6 months old; (F) CL13: 5 months old DEC tissue.

**Additional file 10: Table S6.** Survival of DEC tissues on CIM containing different concentration of geneticin after 8 weeks of culture.

**Additional file 11: Table S7.** Effect of l-cysteine and ascorbic acid on post bombardment recovery of DEC tissues and GUS gene expression.

## Abbreviations

BA: benzylaminopurine
NAA: *a*-naphthalene acetic acid
MS: Murashige and Skoog media
PVP: polyvinylpyrrolidone
KN: kinetin/6-furfurylaminopurine
IAA: indole-3-acetic acid
IBA: indole-3-butyric acid
LA: l-lipoic acid
2,4-D: 2,4-dichlorophenoxyacetic acid
TDZ: thidiazuron
