# Supplementary material for: Robust genetic transformation of sorghum (Sorghum bicolor L.) using differentiating embryogenic callus induced from immature embryos
Source: Plant Methods. 2017 Dec 8;13:109. doi: 10.1186/s13007-017-0260-9 (PMC5723044; doi:10.1186/s13007-017-0260-9)
Supplement: Supplementary file 1 — Additional file 1: Table S1. Composition of media used in different steps of DEC tissue induction and plant regeneration from sorghum. [file 13007_2017_260_MOESM1_ESM.docx]

**Table S1.** Composition of media used in different steps of DEC tissue induction and plant regeneration from sorghum.

| Component | Callus induction  (CIM) | Shoot induction  (SIM) | Shoot Regeneration  (SRM) | Shoot out growth  (SOG) | Root induction  (RIM) |
| --- | --- | --- | --- | --- | --- |
| Basal Medium  (g/l) | MS  4.33 | MS  4.33 | MS  4.33 | MS (Half strength)  2.2 | MS  4.33 |
| 2,4-D (mg/l) | 1.0 | 0.5 | - | - | - |
| BAP (mg/l) | 0.5 | 1.0 | 1.0 | - | - |
| TDZ (mg/l) | - | - | 0.5 | - | - |
| NAA (mg/l) | - | - | - | - | 1.0 |
| IAA (mg/l) | - | - | - | - | 1.0 |
| IBA (mg/l) | - | - | - | - | 1.0 |
| L-proline (g/l) | 0.7 | 0.7 | 0.7 | - | - |
| L-Lipoic acid (mg/l) | 1 | 1 | 1 | 1 | 1 |
| Peptone (g/l) | 0.82 | 0.82 | 0.82 | 0.82 | 0.82 |
| Myo-inositol (g/l) | 0.15 | 0.15 | 0.15 | 0.15 | 0.15 |
| CuSO4 (mg/l) | 0.8 | 0.8 | 0.8 | 0.8 | 0.8 |
| PVP (g/l) | - | - | - | - | 2 |
| Maltose (g/l) | 30 | 30 | 30 | - | - |
| Sucrose (g/l) | - | - | - | 15 | 15 |
| Agar (Type A) (g/l) | 4.5 | 4.5 | 4.5 | 4.5 | 4.5 |
| pH | 5.8 | 5.8 | 5.8 | 5.8 | 5.8 |
