# Supplementary material for: Robust genetic transformation of sorghum (Sorghum bicolor L.) using differentiating embryogenic callus induced from immature embryos
Source: Plant Methods. 2017 Dec 8;13:109. doi: 10.1186/s13007-017-0260-9 (PMC5723044; doi:10.1186/s13007-017-0260-9)
Supplement: Supplementary file 2 — Additional file 2: Table S2. Composition of media used in different steps of sorghum transformation using DEC tissues by particle bombardment. [file 13007_2017_260_MOESM2_ESM.docx]

**Table S2.** Composition of media used in different steps of sorghum transformation using DEC tissues by particle bombardment.

| Component | Osmotic medium  (CIM-OS) | Pre-selection  Medium  (CIM-PS) | Callus induction  (CIM-G25) | Shoot induction  (SIM-G35) | Shoot Regeneration  (SRM-G25) | Shoot out growth  (SOG-G15) | Root induction  (RIM-G15) |
| --- | --- | --- | --- | --- | --- | --- | --- |
| MS ( g/l) | 4.33 | 4.33 | 4.33 | 4.33 | 4.33 | 2.2 | 4.33 |
| ***Growth regulators*** | | | | | | | |
| 2,4-D (mg/l) | 1.0 | 1.0 | 1.0 | 0.5 | - | - | - |
| BAP (mg/l) | 0.5 | 0.5 | 0.5 | 1.0 | 1.0 | - | - |
| TDZ (mg/l) | - | - | - | - | 0.5 | - | - |
| NAA (mg/l) | - | - | - | - | - | - | 1.0 |
| IAA (mg/l) | - | - | - | - | - | - | 1.0 |
| IBA (mg/l) | - | - | - | - | - | - | 1.0 |
| ***Amino acids*** | | | | | | | |
| L-proline (g/l) | 0.7 | 0.7 | 0.7 | 0.7 | 0.7 | - | - |
| ***Antioxidants*** | | | | | | | |
| L-Lipoic acid (mg/l) | 1 | 1 | 1 | 1 | 1 | 1 | 1 |
| L-cysteine (mg/l) | - | 50 | - | - | - | - | - |
| Ascorbic acid (mg/l) | - | 15 | - | - | - | - | - |
| ***Others*** | | | | | | | |
| Peptone (g/l) | 0.82 | 0.82 | 0.82 | 0.82 | 0.82 | 0.82 | 0.82 |
| Myo-inositol | 0.15 | 0.15 | 0.15 | 0.15 | 0.15 | 0.1 | 0.15 |
| CuSO4 (mg/l) | 0.8 | 0.8 | 0.8 | 0.8 | 0. | 0.8 | 0.8 |
| PVP (g/l) | - | - | - | - | - | - | 2 |
| ***Sugars*** | | | | | | | |
| Maltose (g/l) | - | 30 | 30 | 30 | 30 | - | - |
| Sucrose (g/l) | - | - | - | - | - | 15 | 15 |
| Mannitol (g/l) | 36.4 | - | - | - | - | - | - |
| Sorbitol (g/l) | 36.4 | - | - | - | - | - | - |
| ***Antibiotics*** |  | | | | | | |
| Geneticin (mg/l) | - | - | 25 | 30 | 25 | 15 | 15 |
| ***Solidifying agent*** | | | | | | | |
| Type A agar (g/l) | 4.25 | 4.5 | 4.5 | 4.5 | 4.5 | 4.5 | 4.5 |
| pH | 5.8 | 5.8 | 5.8 | 5.8 | 5.8 | 5.8 | 5.8 |
| Culture deration | 24 hrs (4 prior bombardment and 18 hrs after bombardment | 3-4 days | 4 weeks | 2 weeks | 2 weeks | 2 weeks | 2-3 weeks |
