# Supplementary figures and images for: Robust genetic transformation of sorghum (Sorghum bicolor L.) using differentiating embryogenic callus induced from immature embryos
Source: Plant Methods. 2017 Dec 8;13:109. doi: 10.1186/s13007-017-0260-9 (PMC5723044; doi:10.1186/s13007-017-0260-9)

## Slide 1
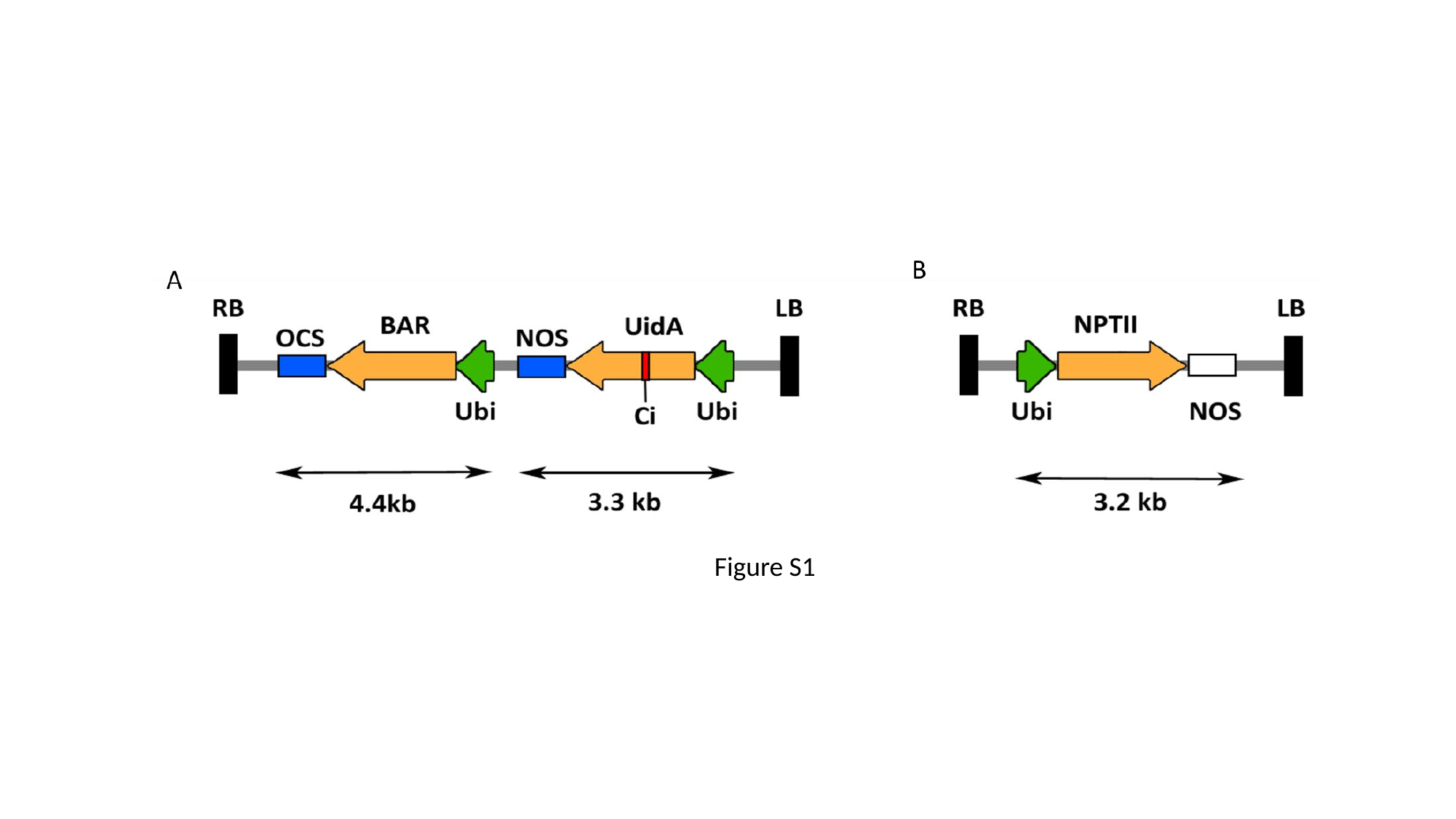

Figure S1

Supplement: Supplementary file 3 — Additional file 3: Figure S1. Schematic representation of plasmids used for bombardment of sorghum DEC tissue. A-pUbi-BAR, B-pBSV003. [file 13007_2017_260_MOESM3_ESM.pptx]

## Slide 1
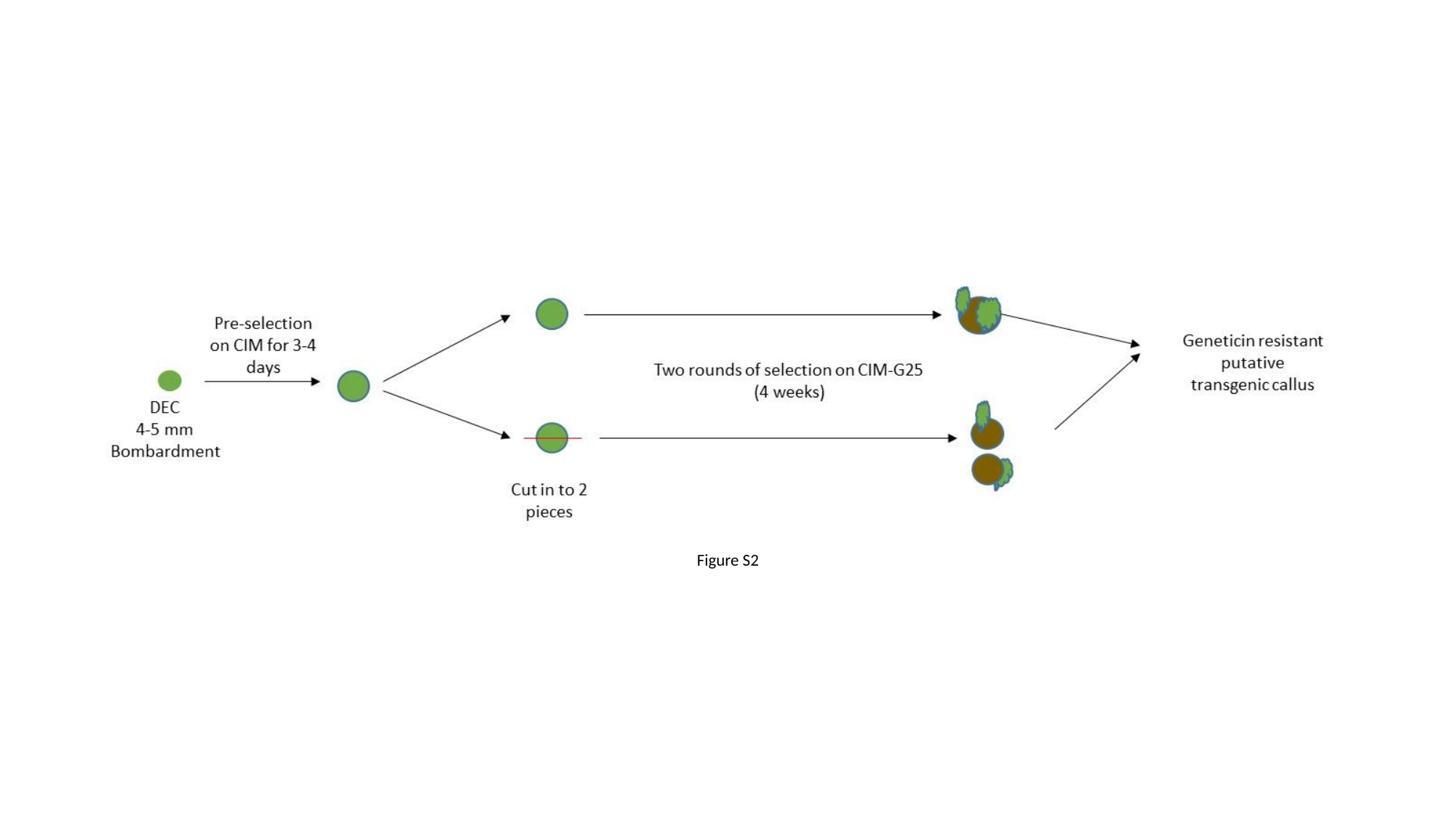

Figure S2

Supplement: Supplementary file 4 — Additional file 4: Figure S2. Method of sectioning and culturing bombarded DEC tissue in selection media. [file 13007_2017_260_MOESM4_ESM.pptx]

## Slide 1
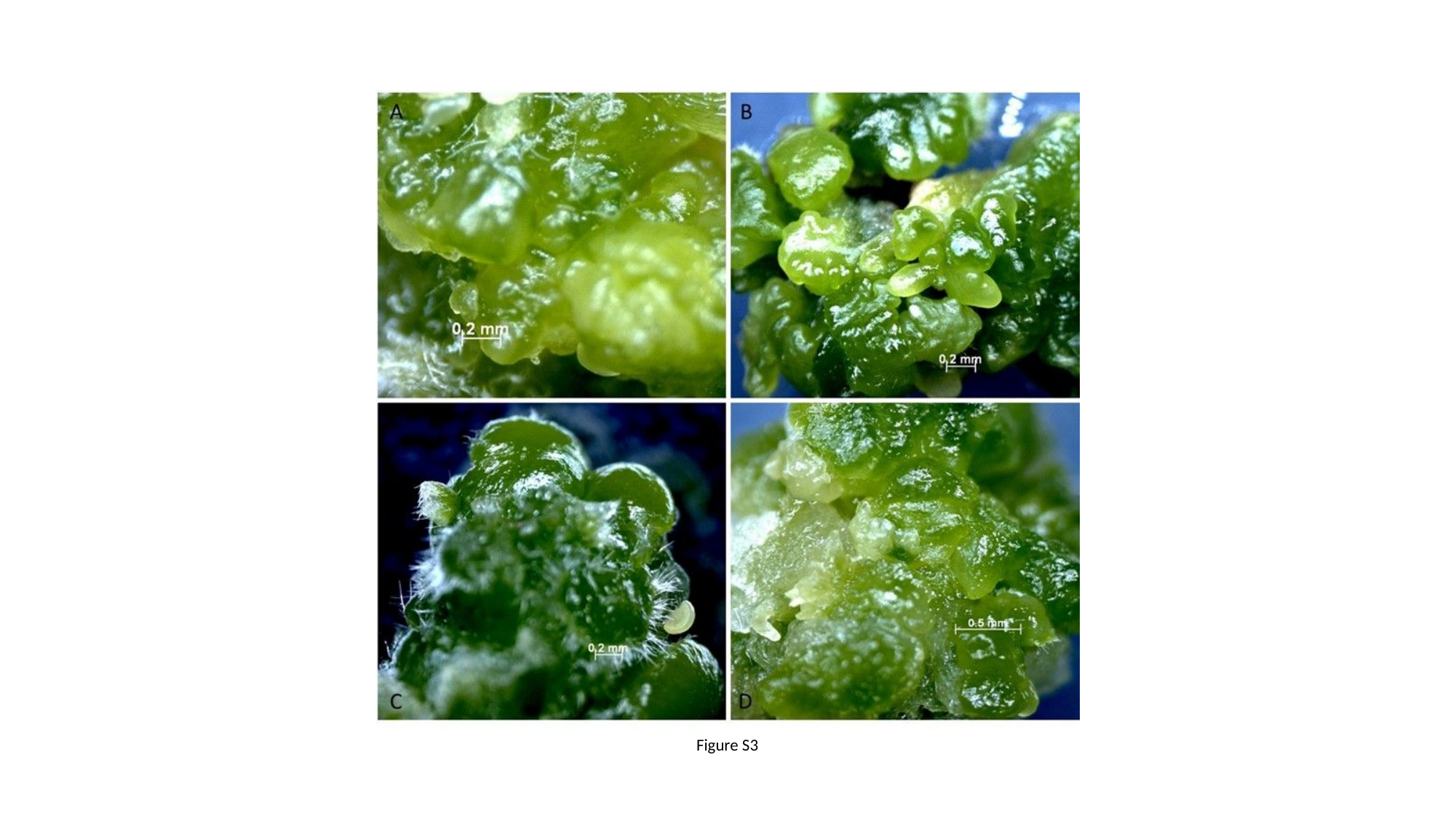

Figure S3

Supplement: Supplementary file 7 — Additional file 7: Figure S3. Stages of differentiating embryogenic callus (DEC) induced and maintained on CIM. A, B Initiation of globular stages embryos. C, D Enlargement of globular stages embryos with out further differentiation. [file 13007_2017_260_MOESM7_ESM.pptx]

## Slide 1
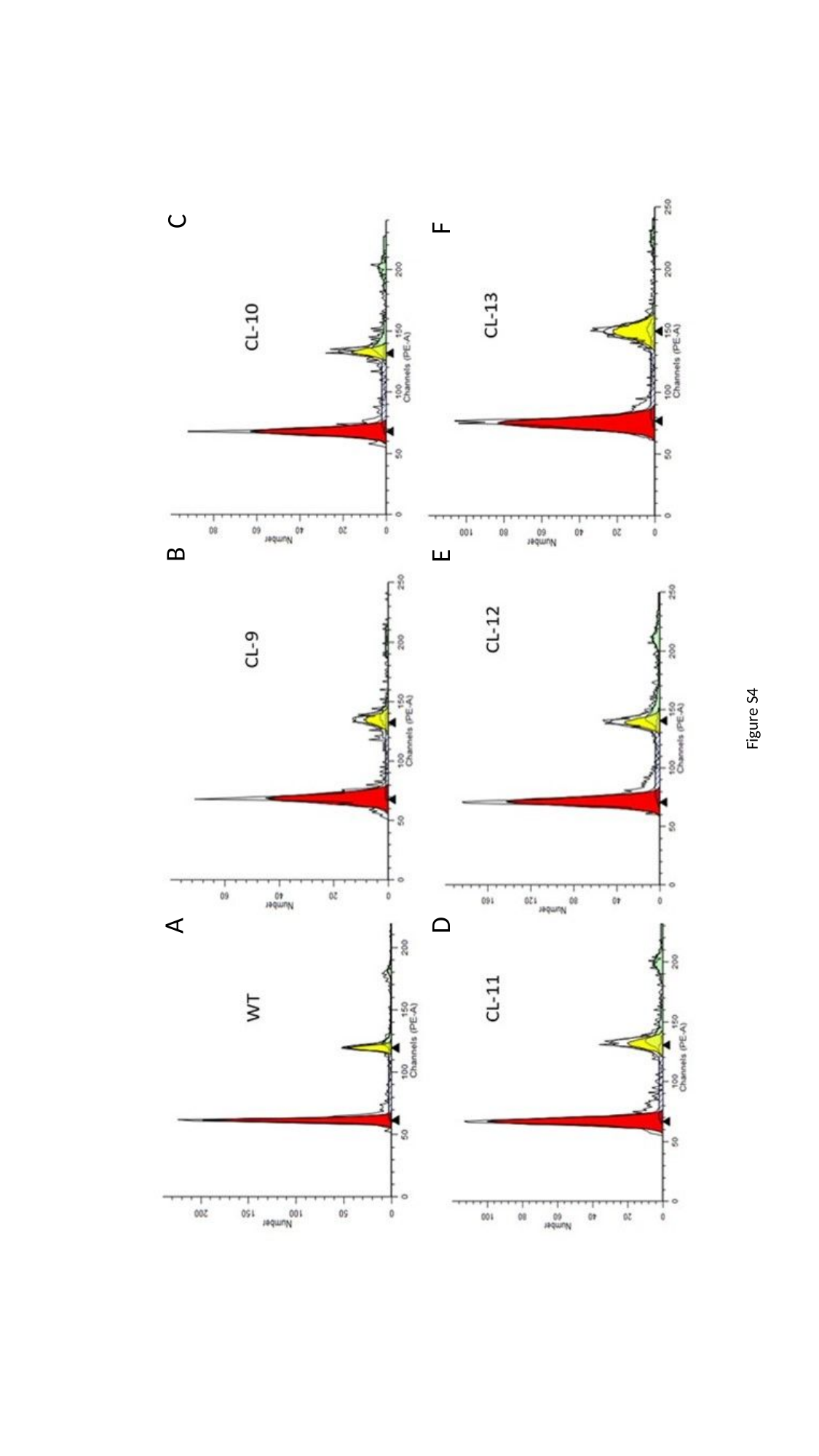

Figure S4
A
B
C
D
E
F

Supplement: Supplementary file 9 — Additional file 9: Figure S4. Histograms of nuclei extracted from leaf tissue of regenerated plants from different age callus lines and WT seedling. (A) WT leaf tissue from 3 weeks old plant; (B) CL9: 24 months; (C) CL10: 12 months old; (D-E) CL-11 and CL12: 6 months old; (F) CL13: 5 months old DEC tissue. [file 13007_2017_260_MOESM9_ESM.pptx]
