# Supplementary material for: Robust genetic transformation of sorghum (Sorghum bicolor L.) using differentiating embryogenic callus induced from immature embryos
Source: Plant Methods. 2017 Dec 8;13:109. doi: 10.1186/s13007-017-0260-9 (PMC5723044; doi:10.1186/s13007-017-0260-9)
Supplement: Supplementary file 5 — Additional file 5: Table S3. List of primers and probes used in copy number detection by ddPCR in transgenic sorghum. [file 13007_2017_260_MOESM5_ESM.docx]

**Table S3.** List of primers and probes used in copy number detection by ddPCR in transgenic sorghum

| Gene ID | Primer sequence | | Probe sequence |
| --- | --- | --- | --- |
|  | Forward | Reverse |  |
| *NPTII* | 5’-tacgcttgatccggctac-3’ | 5’-cttccatccgagtacgtg-3’ | gaaacatcgcatcgagcg |
| *ENOL*-2 | 5’-tgaggacccttttgatcagg-3’ | 5’-caagccttcttgccaatagc-3’ | tggagttcatgggcatcattgca |
