# Supplementary material for: Robust genetic transformation of sorghum (Sorghum bicolor L.) using differentiating embryogenic callus induced from immature embryos
Source: Plant Methods. 2017 Dec 8;13:109. doi: 10.1186/s13007-017-0260-9 (PMC5723044; doi:10.1186/s13007-017-0260-9)
Supplement: Supplementary file 6 — Additional file 6: Table S4. Effect of LA on DEC tissue yield from 45 immature embryos (1.4–2.0 mm) of sorghum. [file 13007_2017_260_MOESM6_ESM.docx]

**Table S4.** Effect of LA on DEC tissue yield from 45 immature embryos (1.4 to 2.0 mm) of sorghum

| Culturing time (weeks) | DEC yield in number (~5 mm each and ready to use) | |
| --- | --- | --- |
|  | CIM + 1 mg/l LA | CIM without LA |
| 4 | 36^A^ | 29^a^ |
| 6 | 90^B^ | 45^b^ |
| 8 | 120^C^ | 45^c^ |
| 10 | 218^D^ | 150^d^ |
| 12 | 825^E^ | 435^e^ |

Different case letters in the row are statistically significant by pairwise comparison
