# Supplementary material for: Robust genetic transformation of sorghum (Sorghum bicolor L.) using differentiating embryogenic callus induced from immature embryos
Source: Plant Methods. 2017 Dec 8;13:109. doi: 10.1186/s13007-017-0260-9 (PMC5723044; doi:10.1186/s13007-017-0260-9)
Supplement: Supplementary file 8 — Additional file 8: Table S5. 2C values of nuclear DNA content of leaf tissue regenerated from different age callus lines. [file 13007_2017_260_MOESM8_ESM.docx]

**Table S5.** 2C values of nuclear DNA content of leaf tissue regenerated from different age callus lines.

| Number of the callus line | Age of the callus line from induction (months) | nDNA content (pg) |
| --- | --- | --- |
| CL-09 | 24 | 1.72 +0.014^a^ |
| CL-10 | 12 | 1.71+ 0.028 ^a^ |
| CL-11 | 6 | 1.78+ 0.169 ^a^ |
| CL-12 | 6 | 1.76+0.007 ^a^ |
| CL-13 | 5 | 1.98+0.169 ^*^ |
| WT | | 1.67 pg |

Values are the means with standard deviation (SD).

^a^ Each treatment has 2 plants with 2 replications. Means were compared using the Multiple Pair wise Comparison Procedure and found to be not significantly different (CL-9 to CL-12) at P=0.19 with WT sorghum 2C DNA. * Significantly different to WT (P=0.034).
