# Supplementary material for: Robust genetic transformation of sorghum (Sorghum bicolor L.) using differentiating embryogenic callus induced from immature embryos
Source: Plant Methods. 2017 Dec 8;13:109. doi: 10.1186/s13007-017-0260-9 (PMC5723044; doi:10.1186/s13007-017-0260-9)
Supplement: Supplementary file 10 — Additional file 10: Table S6. Survival of DEC tissues on CIM containing different concentration of geneticin after 8 weeks of culture. [file 13007_2017_260_MOESM10_ESM.docx]

**Table S6.** Survival of DEC tissues on CIM containing different concentration of geneticin after 8 weeks of culture.

| Media | Geneticin concentration (mg/l) | DEC tissue survival (%) |
| --- | --- | --- |
| CIM (Table S1) | 0 | 100 ± 0.0 |
|  | 15 | 80 ± 1.0 |
|  | 25 | 4 ± 2.6 |
|  | 35 | 2 ± 0.5 |
|  | 45 | 0 |

Values are the means with standard deviation (SD)
