# Supplementary material for: Robust genetic transformation of sorghum (Sorghum bicolor L.) using differentiating embryogenic callus induced from immature embryos
Source: Plant Methods. 2017 Dec 8;13:109. doi: 10.1186/s13007-017-0260-9 (PMC5723044; doi:10.1186/s13007-017-0260-9)
Supplement: Supplementary file 11 — Additional file 11: Table S7. Effect of l-cysteine and ascorbic acid on post bombardment recovery of DEC tissues and GUS gene expression. [file 13007_2017_260_MOESM11_ESM.docx]

**Table S7.** Effect of L-cysteine and ascorbic acid on post bombardment recovery of DEC tissues and GUS gene expression

| Number of tissues bombarded | Number of brown tissues* | | Number of GUS spots/tissue** | |
| --- | --- | --- | --- | --- |
|  | CIM no L-cysteine and ascorbic acid | CIM + L-cysteine and ascorbic acid | CIM no L-cysteine and ascorbic acid | CIM + cysteine and ascorbic acid |
| Exp-A: 75 | 15 | 8 | 12 | 25 |
| Exp-B: 75 | 18 | 11 | 13 | 20 |
| Exp-C: 75 | 23 | 14 | 7 | 38 |
| Average | 18.6 ± 4.04 | 11.0 ± 4.04 | 10.6 ± 3.2 | 27.6 ± 9.2 |

Values are the means with standard deviation (SD). *Not significant (P=0.083); ** Significant (P=0.039)
